# Supplementary material for: Increased Gene Expression of C1orf74 Is Associated with Poor Prognosis in Cervical Cancer
Source: Cells. 2023 Oct 27;12(21):2530. doi: 10.3390/cells12212530 (PMC10649411; doi:10.3390/cells12212530)
Supplement: Supplementary file 1 [file cells-12-02530-s001.zip › cells-2646325-SM.pdf]

Supplementary Materials

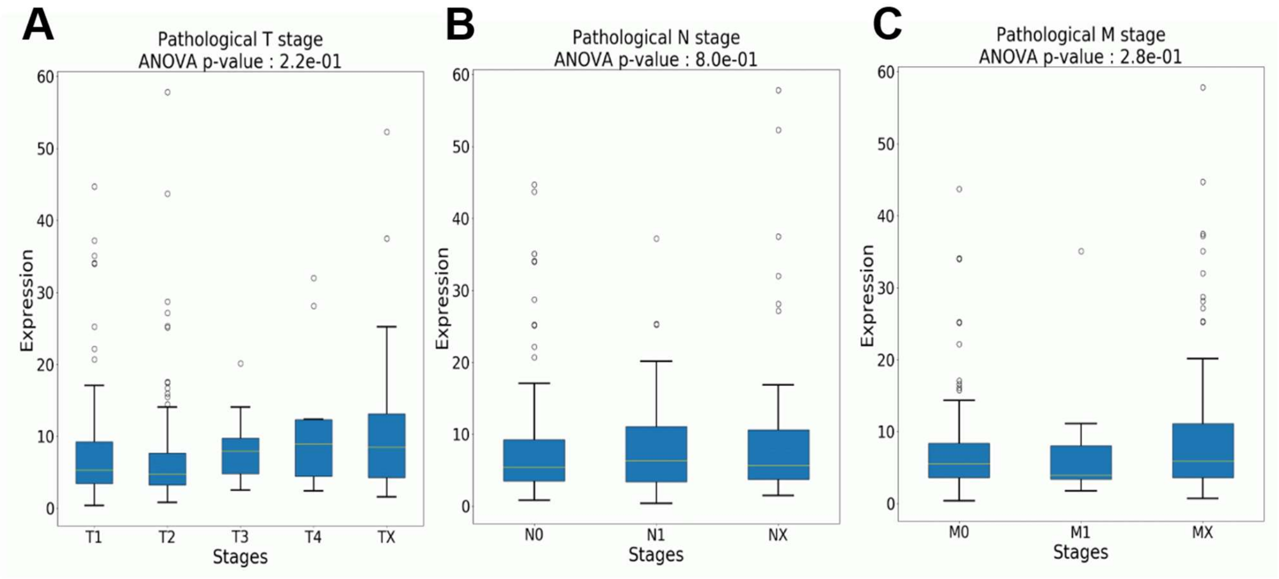

Figure S1: (A) C1orf74 expression at Pathological T stages, (B) C1orf74 expression at Pathological N stages , (A) C1orf74 expression at Pathological M stages

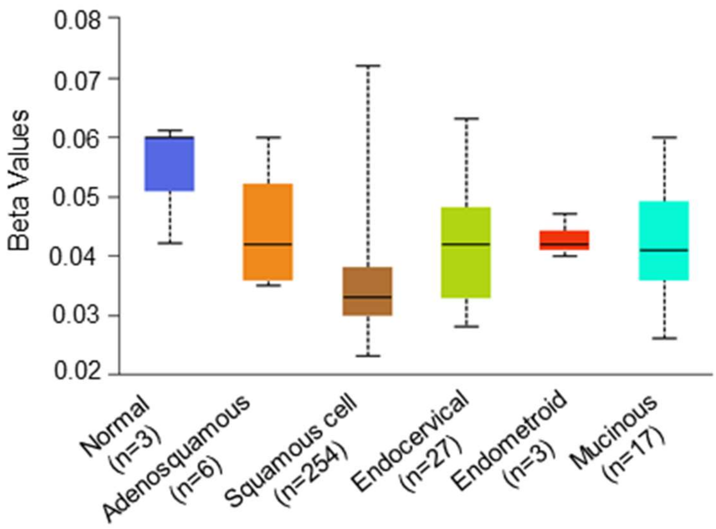

Figure S2: Promoter methylation levels of C1orf74 gene across different histological types of cervical cancer (UALCAN) **(***p < 0.05, ** p < 0.01, *** p < 0.001**,** **** p <0.0001, ns= not significant). p-values are mentioned in table S3.

Table S1. Statistical significance (p-values) of expression levels of C1orf74 in cervical cancer.

**Statistical significance: Expression of C1orf74 in CESC based on sample type (TNMplot)**	
**Comparison**	Statistical significance

Normal-Tumor	1.56E-03
Normal-Metastatic	9.44E-03
Tumor-Metastatic	2.73E-01

Statistical significance: Expression of C1orf74 in CESC based on cancer stages (UALCAN)

Comparison	Statistical significance
Normal-vs-Stage1	7.33E-15
Normal-vs-Stage2	6.96E-09
Normal-vs-Stage3	6.59E-09
Normal-vs-Stage4	1.37E-04
Stage1-vs-Stage2	4.31E-01
Stage1-vs-Stage3	3.82E-02
Stage1-vs-Stage4	2.87E-01
Stage2-vs-Stage3	2.42E-01
Stage2-vs-Stage4	7.75E-01
Stage3-vs-Stage4	5.33E-01

Statistical significance: Expression of C1orf74 in CESC based on tumor histology (UALCAN)

Comparison	Statistical significance
Adenosquamous-VS-Squamous-cell	1.84E-08
Adenosquamous-VS-Endocervical	3.94E-01
Adenosquamous-VS-Endometrioid	6.33E-01
Adenosquamous-VS-Mucinous	7.20E-01
Adenosquamous-VS-Normal	4.12E-03
Squamous-cell-vs-Endocervical	<1E-12
Squamous-cell-vs-Endometrioid	5.68E-07
Squamous-cell-vs -Mucinous	1.12E-11
Squamous-cell-vs-Normal	1.62E-12
Endocervical-vs-Endometrioid	2.86E-01
Endocervical-vs-Mucinous	7.78E-02
Endocervical-vs-Normal	1.13E-02
Endometrioid-vs-Mucinous	9.86E-01
Endometrioid-vs-Normal	2.62E-03
Mucinous-vs-Normal	1.00E-02

Statistical significance: Expression of C1orf74 in CESC based on HPV Positivity (OncoDB)

Comparison	Statistical significance
HPV positive vs HPV negative	3.90E-04

Table S2. Statistical significance (p-values) of expression levels of C1orf74 in cervical cancer cell line and patients.

Statistical significance: Expression of C1orf74 in cervical cancer positive cell lines

Comparison	Statistical significance
Control vs HeLa	0.0003
Control vs SiHa	0.0001
Control vs CasKi	0.0094
Control vs C33A	0.3982

Statistical significance: Expression of C1orf74 in cervical cancer patients in the study	
Comparison	Statistical significance
Control vs Patient 14	0.0003
Control vs Patient 13	0.2378
Control vs Patient 6	0.0021
Control vs Patient 7	<0.0001
Control vs Patient 16	<0.0001
Control vs Patient 19	<0.0001
Control vs Patient 23	0.1369
Control vs Patient 26	0.0001
Control vs Patient 28	<0.0001
Control vs Patient 31	<0.0001
Control vs Patient 34	<0.0001
Control vs Patient 36	0.0001
Control vs Patient 43	0.0013
Control vs Patient 47	<0.0001
Control vs Patient 54	0.006
Control vs Patient 51	<0.0001
Control vs Patient 10	0.0061
Control vs Patient 15	<0.0001
Control vs Patient 17	<0.0001
Control vs Patient 24	0.0163
Control vs Patient 40	0.0003
Control vs Patient 45	0.004
Control vs Patient 11	0.038
Control vs Patient 18	0.0008
Control vs Patient 56	0.2895

Table S3. Statistical significance (p-values) of promoter methylation levels of C1orf74 in cervical cancer.

**Statistical significance: Promoter Methylation levels of C1orf74 in CESC (UALCAN)**	
Comparison	Statistical significance
Normal-vs-Primary	5.58E-03
**Statistical significance: Promoter Methylation levels of C1orf74 based on cancer stages in CESC**	
Comparison	Statistical significance
Normal-vs-Stage1	9.81E-02
Normal-vs-Stage2	3.34E-02
Normal-vs-Stage3	1.66E-02
Normal-vs-Stage4	1.55E-01
Stage1-vs-Stage2	2.53E-01
Stage1-vs-Stage3	9.92E-02
Stage1-vs-Stage4	3.34E-01
Stage2-vs-Stage3	5.11E-01
Stage2-vs-Stage4	6.70E-01
Stage3-vs-Stage4	9.54E-01
**Statistical significance: Promoter Methylation levels of C1orf74 base on tumor histology in CESC (UALCAN)**	

Comparison	Statistical significance
Normal-VS-Adenosquamous	4.42E-01
Normal-VS-Squamous	1.36E-03
Normal-VS-Endocervical	9.13E-02
Normal-VS-Endometrioid	1.61E-01
Normal-VS-Mucinous	6.94E-02
Adenosquamous-VS-Squamous	8.95E-01
Adenosquamous-VS-Endocervical	3.50E-01
Adenosquamous-VS-Endometrioid	3.48E-01
Adenosquamous-VS-Mucinous	3.45E-01
Squamous-VS-Endocervical	8.50E-09
Squamous-VS-Endometrioid	1.17E-08
Squamous-VS-Mucinous	7.36E-09
Endocervical-VS-Endometrioid	9.69E-01
Endocervical-VS-Mucinous	8.12E-01
Endometrioid-VS-Mucinous	9.30E-01

Table S4. Genes positively correlated with C1orf74 in CESC based on UALCAN web portal.

SL No.	Genes	Pearson CC
1	IRF6	0.61
2	PPP1R13L	0.53
3	RAET1G	0.5
4	TUBB6	0.43
5	DSE	0.42
6	CDH3	0.42
7	C16orf74	0.41
8	KIAA1609	0.4
9	KRT17	0.4
10	CHRNB1	0.39
11	EFHD2	0.38
12	LUZP1	0.38
13	NTF4	0.38
14	SHC1	0.38
15	TPM3	0.38
16	G0S2	0.38
17	ITGA3	0.37
18	RPS6KA4	0.37
19	TMEM22	0.37
20	TBC1D2	0.37
21	RIN1	0.37
22	DFNA5	0.37
23	SLC2A9	0.37
24	SYDE1	0.37
25	LOC728643	0.37

Table S5: list of genes whose expression levels are significantly differential between patients stratified by clinical parameters in cervical cancer
